# Repulsive parallel MCMC algorithm for discovering diverse motifs from large sequence sets

**DOI:** 10.1093/bioinformatics/btv017

**Published:** 2015-01-11

**Authors:** Hisaki Ikebata, Ryo Yoshida

**Affiliations:** ^1^Department of Statistical Science, The Graduate University for Advanced Studies (Sokendai), 10-3 Midori-cho, Tachikawa, Tokyo 190-8562, Japan, ^2^Department of Statistical Modeling, The Institute of Statistical Mathematics, Research Organization of Information and Systems, 10-3 Midori-cho, Tachikawa, Tokyo 190-8562, Japan, ^3^JST-CREST, 10-3 Midori-cho, Tachikawa, Tokyo 190-8562, Japan, ^4^JST-ERATO Sato Live Bio-Forecasting Project, 2-2-2 Hikaridai Seika-cho, Soraku-gun, Khoto-fu 619-0288, Japan and ^5^The Thomas N. Sato BioMEC-X Laboratories, Advanced Telecommunications Research Institute International, 2-2-2 Hikaridai Seika-cho, Soraku-gun, Khoto-fu 619-0288, Japan

## Abstract

**Motivation:** The motif discovery problem consists of finding recurring patterns of short strings in a set of nucleotide sequences. This classical problem is receiving renewed attention as most early motif discovery methods lack the ability to handle large data of recent genome-wide ChIP studies. New ChIP-tailored methods focus on reducing computation time and pay little regard to the accuracy of motif detection. Unlike such methods, our method focuses on increasing the detection accuracy while maintaining the computation efficiency at an acceptable level. The major advantage of our method is that it can mine *diverse multiple* motifs undetectable by current methods.

**Results:** The repulsive parallel Markov chain Monte Carlo (RPMCMC) algorithm that we propose is a parallel version of the widely used Gibbs motif sampler. RPMCMC is run on parallel interacting motif samplers. A repulsive force is generated when different motifs produced by different samplers near each other. Thus, different samplers explore different motifs. In this way, we can detect much more diverse motifs than conventional methods can. Through application to 228 transcription factor ChIP-seq datasets of the ENCODE project, we show that the RPMCMC algorithm can find many reliable cofactor interacting motifs that existing methods are unable to discover.

**Availability and implementation:** A C++ implementation of RPMCMC and discovered cofactor motifs for the 228 ENCODE ChIP-seq datasets are available from http://daweb.ism.ac.jp/yoshidalab/motif.

**Contact:** ikebata.hisaki@ism.ac.jp, yoshidar@ism.ac.jp

**Supplementary information:** Supplementary data are available from *Bioinformatics* online.

## 1 Introduction

The motif discovery problem has been receiving renewed attention since recent experimental technologies, such as ChIP-seq, posed new challenges. The problem is to identify recurring patterns of conserved short strings that appear in a large fraction of nucleotide sequences. A genome-wide ChIP study produces thousands or more DNA fragments consisting of several hundred base pairs, which cover the binding sites of a transcription factor (TF). By discovering motifs in the given sequences, which are associated with known TF-binding motifs in a database, e.g. JASPAR (Sandelin *et* *al*., 2004), TRANSFAC (Wingender *et* *al*., 1995), we can predict not only the regions bound by the primary TF but also the cofactors that modulate the TF activity (Bailey, 2011; Goi *et* *al*., 2013; Smith *et* *al*., 2005).

Early methods of *de novo* motif discovery can be classified into either a model-based [MEME (Bailey and Elkan, 1994), AlignACE (Hughes *et* *al*., 2000), ANN-Spec (Workman and Stormo, 2000)] or a word-count approach [Weeder (Pavesi *et* *al*., 2001)]. These methods were designed on the assumption that the input sequences of ∼10^3^ base pairs would range in size from 10^2^ to 10^3^. Hence, they do not scale to the size of ChIP-seq data and their fundamental methodologies have undergone reconstruction. However, most ChIP-tailored algorithms emphasize computational efficiency, and they sacrifice accuracy of motif detection because they use heuristics to speed up their computation time.

The model-based methods employ either the EM algorithm (Bailey and Elkan, 1994) or Gibbs sampling (Lawrence *et* *al*., 1993). The main computational load arises in the process of calculating the posterior probabilities over all fixed-length subsequences at every iteration. STEME (Reid and Wernisch, 2011), a ChIP-tailored version of MEME, uses a branch-and-bound technique, so that negligible oligomers with significantly low probabilities are effectively removed. The word-count methods, regardless of old or new, rely on essentially the same strategy. All possible oligomers are counted with exact or the fuzzy matching for input sequences. Then, overrepresented oligomers are determined against background sequences. Similar motifs are merged to generate output motifs. To reduce the computational load in the counting operation, DREME (Bailey, 2011) and CisFinder (Sharov and Ko, 2009) adopt similar strategies. Starting from ≃100 oligomers with no wildcards, each oligomer is either left or removed recursively by adding a wildcard and by assessing its significance. Such methods run the risk of missing important motifs in earlier steps of the recursion. Hegma (Ichinose *et* *al*., 2012) is the fastest of current algorithms. A highly specific strategy based on Gray codes (Gray, 1947) is employed to avoid fuzzy matching so as to speed up the merging of similar motifs. However, this novel idea results in a degradation of the detection accuracy as will be shown later.

The aim of this study is to derive a new algorithm that achieves high detection accuracy while maintaining the computational efficiency at an acceptable level. In particular, the proposed method is designed to detect *many diverse* motifs that previous methods are unable to discover. The proposed repulsive parallel MCMC (RPMCMC) algorithm is a parallel version of the widely used Gibbs motif sampler. One critical drawback of the standard Gibbs sampling, as with the EM algorithm, arises from the following fact: the posterior distribution is highly multimodal because many diverse motifs are present in given sequences. Once the generated Markov chain is absorbed to a locally high probability region, it is difficult to escape from that region within a finite time. This problem has received little attention in previous studies. MEME adopts a serial implementation of the EM algorithm that repeats the search with different initial conditions (Bailey and Elkan, 1994). To reduce the possibility of becoming trapped in the same local optima, low prior probabilities are assigned to already-discovered motif sites in consecutive serial runs. However, such iterative methods take too long to be used for large ChIP data.

RPMCMC is run on parallel interacting Gibbs samplers. A repulsive force comes into play when the trajectories of different chains near each other. Therefore, different chains are facilitated to explore different regions. Compared with the original method using a single chain, this all-at-once interacting parallel run can detect much more diverse motifs. In addition, the proposed method has other unique characteristics, for instance automated control of variable-length motifs, and the fast-clustering algorithm for many generated motifs in the summarization step. We implemented the RPMCMC algorithm with C++, which is available from the Supplementary Website. The method was comprehensively tested on synthetic promoter sequences and 228 TF ChIP-seq datasets of the ENCODE project. In the synthetic promoter analysis, RPMCMC found around 1.5 times as many embedded motifs as existing methods did. For the ChIP-seq datasets, the RPMCMC algorithm reported 444 reliable cofactors in total, 219 of which were not discovered by either of the recently published ChIP-tailored algorithms: DREME and Hegma. On the Supplementary Website, we provide all the discovered cofactor motifs which were associated with annotated motifs in JASPAR.

## 2 Methods

### 2.1 Model

We use the ZOOPS model (Bailey and Elkan, 1994) that allows zero or one motif occurrence per sequence. Assume that we are given a set of *n* sequences, $S^{+}=\{s_{1}^{+},\ldots ,s_{n}^{+}\}$, where sequence $s_{i}^{+}$ is of length *L_i_* (i=1,…,n). The reverse complement of the given sequence set is denoted by $S^{-}=\{s_{1}^{-},\ldots ,s_{n}^{-}\}$. Our model uses the set of *n* concatenated sequences, $S=\{s_{1},\ldots ,s_{n}\}$, where $s_{i}=(s_{i}^{+},s_{i}^{-})$ (i=1,…,n). The motif presence indicator *z_i_* takes the value 1 or 0 according to the presence or absence of a motif in sequence *s_i_*. In a sequence *s_i_* with *z_i_* = 1, a *K*-mer motif is positioned at the start site $u_{i}\in \{1,\ldots ,L_{i}-K+1,L_{i}+1,\ldots ,2L_{i}-K+1$}. The *k*th element of the motif follows the position-specific multinomial distribution with $\theta_{k}={\left(\theta_{k,a},\theta_{k,c},\theta_{k,g},\theta_{k,t}\right)}^{\text{T}}$, which represents the nucleotide preference of the *k*th element to A, C, G, T. Thus, we have $\Theta =(\theta_{1},\ldots ,\theta_{K})$, a position probability matrix (PPM). We treat the motif length *K* as an unknown parameter. The background sequences are assumed to follow independent multinomial trials with the background probability denoted by $\theta_{0}={\left(\theta_{0,a},\theta_{0,c},\theta_{0,g},\theta_{0,t}\right)}^{\text{T}}$.

Given an input *S*, the objective is to estimate the PPM Θ with the unknown motif length *K* and the background probability θ_0_ where the latent variables comprise $U=\{u_{1},\ldots ,u_{n}\}$ and $Z=\{z_{1},\ldots ,z_{n}\}$. The likelihood is then

(1) p(S|U,Z,K,Θ,θ0)∝∏σ∈{a,c,g,t}θ0,σ∑i=1n∑j=12LiI(si,j=σ)  ×∏k=1K∏σ∈{a,c,g,t}(θk,σθ0,σ)∑i=1nziI(si,ui+k−1=σ),

where $s_{i,j}$ denotes the types of bases at the *j*th position in *s_i_*, and I(·) is the indicator function. The first component of the right-hand side in the first line is the probability of all letters in the *n* input sequences, which is calculated under the background multinomial distribution. The second component is the likelihood ratio that assesses overrepresentation of the *K*-mer segmented sequences against the background.

As the priors on the multinomial parameters, we use the Dirichlet distributions

p(Θ|K)∝∏k=1K∏σ∈{a,c,g,t}(θk,σ)βk,σ−1,p(θ0)∝∏σ∈{a,c,g,t}(θ0,σ)ασ−1,p(K=j)=I(Kmin≤j≤Kmax)Kmax−Kmin+1,

where $\beta_{k}={\left(\beta_{k,a},\beta_{k,c},\beta_{k,g},\beta_{k,t}\right)}^{\text{T}}$ (k=1,…,K) and $\alpha ={\left(\alpha_{a},\alpha_{c},\alpha_{g},\alpha_{t}\right)}^{\text{T}}$ are the concentration parameters fixed at set values. The prior on Θ is conditioned by the motif length *K*. The equal probabilities are assigned to any *K* with a range between the predetermined minimum and maximum motif lengths, $K_{\min}$ and $K_{\max}$.

To complete the joint posterior of all the unknown parameters, we prescribe the priors on *U* and *Z* as follows:

p(ui=u|K)=12(Li−K+1) for i=1,…,n,p(zi=1|K)=γK for i=1,…,n.

The start site *u_i_* of a motif occurs with equal probability in all the possible positions in sequence *s_i_*. The motif presence indicator *z_i_* follows the binomial distribution with the success probability γ*^K^* for each *i* (0≤γ≤1).

Note that although a specific type of modeling is presented here, our current program allows for a certain amount of flexibility in the model specification. For instance a user can choose a higher-order Markov background model up to third order (da Fonseca *et* *al*., 2008) and a position specific prior for the motif start sites (Bailey *et* *al*., 2010).

### 2.2 Multiple motifs and local trap

Let *x* denote all the unknowns, U,Z,K,Θ and θ_0_. To obtain an estimate approximately with the posterior p(x|S), we can employ a Gibbs sampling method. However, the Gibbs motif sampler has a serious drawback in that inherent presence of a great many different motifs causes a complex energy landscape of the posterior distribution. In particular, once the trajectory of a Markov chain comes into a locally high-probability region which corresponds to one of the existing motifs, it is difficult to effect a transfer into another region within a finite runtime. The EM algorithm might exhibit the same defects.

**Fig. 1.**
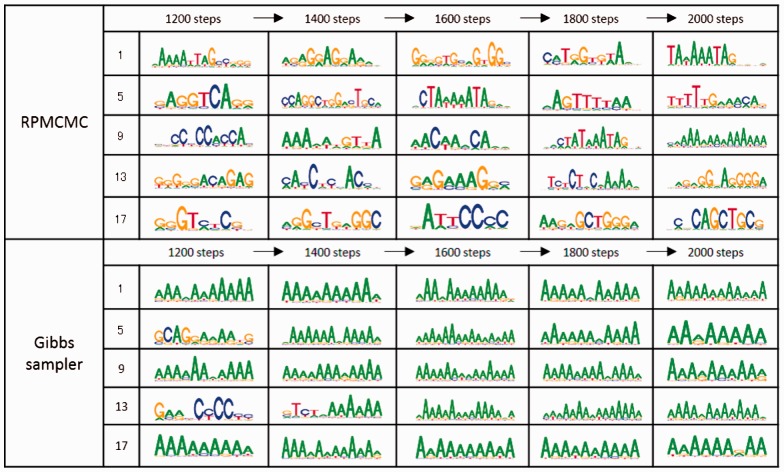
A drawback of the independent Gibbs motif sampler, which is highlighted on 300 promoter sequences. The top and bottom panels display the processes of produced PPMs (sequence-logos) for RPMCMC with 20 replicas and independent Gibbs sampling under 20 different initial conditions. Five of the 20 sampling paths are shown for each method

As an illustration, we show a result of the simple Gibbs motif sampling. The dataset consists of 10^3^-bp-long synthetic promoter sequences of 300 human genes. The Gibbs sampling was repeated 20 times under different initial conditions. As shown in Figure 1, *all* the chains were trapped at similar AT-rich motifs for a long duration. Exceedingly high probabilities might be concentrated on the AT-rich segments and all the chains were absorbed to those domains of the posterior distribution. This is a typical scenario. Figure 1 also shows the result of RPMCMC, which was run with 20 interacting parallel Gibbs samplers as described below. By performing just an all-at-once parallel run, RPMCMC could capture much more diverse motifs than the independent Gibbs sampling could.

### 2.3 RPMCMC algorithm

The RPMCMC algorithm is derived by creating an augmented system $\pi_{A}(x_{1},\ldots ,x_{M}|\beta )$, which consists of *M* exact copies $\pi_{i}(x)=p(x|S)$ (i=1,…,M) of the posterior distribution and the repulsive force function $\psi (x_{1},\ldots ,x_{M})$:

(2) $$\pi_{A}(x_{1},\ldots ,x_{M}|\beta )\propto \prod_{i=1}^{M}\pi (x_{i})\psi {\left(x_{1},\ldots ,x_{M}\right)}^{\beta },\beta \geq 0.$$

**Fig. 2.**
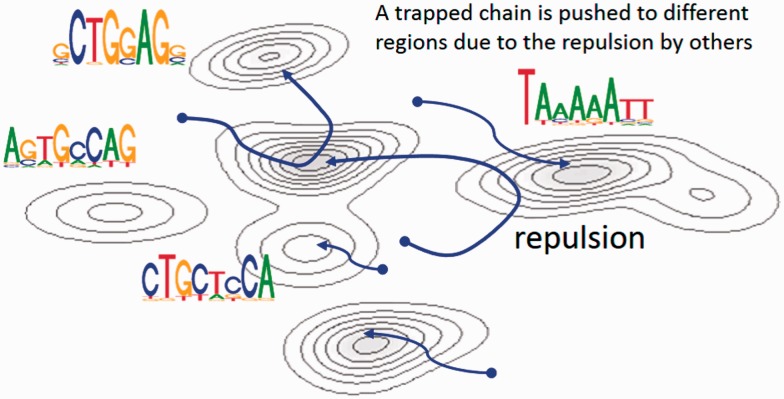
A schematic view of the RPMCMC algorithm

Each *x_i_* is called a *replica*. The repulsive force function ψ imposes a stronger penalty on closer replicas. The parameter β controls the force severity, i.e. a greater β produces a stronger repulsion and vice versa. Drawing samples of $x_{1},\ldots ,x_{M}$ simultaneously from Equation (2), the *M* sample paths tend to move toward different regions. Furthermore, a replica trapped in a locally high probability state can be pushed to other regions by the repulsive force derived from approaching replicas (Fig. 2). It is important to see that the use of a non-zero force severity brings bias to the samples from π*_A_* with respect to the posterior distribution. With β = 0, which removes the repulsion from π*_A_*, an unbiased sample set can be obtained.

The repulsive force function is defined as a function of PPMs, $\psi (x_{1},\ldots ,x_{M})\equiv \psi (\Theta_{1},\ldots ,\Theta_{M})$. Let $D(\Theta_{i},\Theta_{j})$ be an increasing function of the dissimilarity between Θ*_i_* and Θ*_j_*. With this, the repulsion is modeled by

(3) $$\psi (x_{1},\ldots ,x_{M})=\prod_{i=1}^{M}\exp(\underset{j:j<i}{\min}D(\Theta_{i},\Theta_{j})).$$

Replica *i* interacts with its nearest neighbor $j^{*}$, such that $j^{*}=\arg\min_{j:j<i}D(\Theta_{i},\Theta_{j})$. The dissimilarity *D* is measured by

(4) $$\begin{array}{l} D(\Theta_{i},\Theta_{j})=\frac{1}{K^{*}}(\underset{(k,h)\in A}{\min}||\Theta_{i,k:k+K^{*}}-\Theta_{j,h:h+K^{*}}|{|}_{F}\\ +\;c\times |K_{i}-K_{j}|\;), \end{array}$$

where $A=\{(k,h)|k=1,\ldots ,\max(1,K_{j}-K_{i}+1),h=1,\ldots ,\max(1,K_{i}-K_{j}+1)\}$ and $K^{*}=\min\{K_{i},K_{j}\}$. In general, *K_i_* and *K_j_*, the column sizes of Θ*_i_* and Θ*_j_*, are different. The distance of the PPMs is assessed after associating a smaller-sized PPM with the same-sized submatrix of the other, $\Theta_{i,k:k+K^{*}}$ and $\Theta_{j,h:h+K^{*}}$ and by choosing the smallest Frobenius norm in all possible alignments (k,h)∈A. The second term is a gap penalty for the difference of motif lengths where *c* > 0.

To obtain an estimate from the augmented posterior, Gibbs sampling is combined with several techniques such as the reversible-jump MCMC method (Green, 1995) and the slice sampler (Neal, 2003). The full details of the RPMCMC procedure are described in Supplementary Method S1. The proposed method generates Markov chains of the *M* replicas in parallel. Then, different chains move toward different regions of the state space due to the repulsion. We can discover a much wider variety of motifs with an all-at-once interacting parallel simulation than with the independent method. Conventional Gibbs sampling with *M* different initial seeds (as shown in the previous subsection) can be derived by setting the zero force severity, β = 0, to RPMCMC.

Suppose that we are given a sample set of size *N* × *M* from Equation (2) with nonzero β, denoted by $\left\{x_{i}^{\left(j\right)}|i=1,\ldots ,M,j=1,\ldots ,N\right\}$ where each $x_{i}^{\left(j\right)}$ denotes the *j*th sample of the *i*th replica. Obviously, the repulsive force leads to biased samples with respect to the target $\pi_{A}(x_{1},\ldots ,x_{M}|0)$ in Equation (2) at the zero force severity. To correct this bias, the importance sampling is used, which assigns a weight to each sample as

(5) $$w_{i}^{\left(j\right)}=\frac{\pi_{A}(x_{1}^{\left(j\right)},\ldots ,x_{M}^{\left(j\right)}|0)}{\pi_{A}(x_{1}^{\left(j\right)},\ldots ,x_{M}^{\left(j\right)}|\beta )}\propto \frac{1}{\psi (x_{1}^{\left(j\right)},\ldots ,x_{M}^{\left(j\right)})}.$$

The ratio between the target (zero force) and the biased distribution (β>0) becomes the inverse of the repulsive force function. Note that the *M* replicas $x_{i}^{\left(j\right)}$ (i=1,…,M) in the *j*th ensemble share the same weight.

As shown in Supplementary Method S1, our current implementation does not parallelize the process of updating the *M* Markov chains. We use multi-core processors only for counting the nucleotide frequencies when renewing the motif start sites.

### 2.4 Post-processing: clustering and ranking

**Fig. 3.**
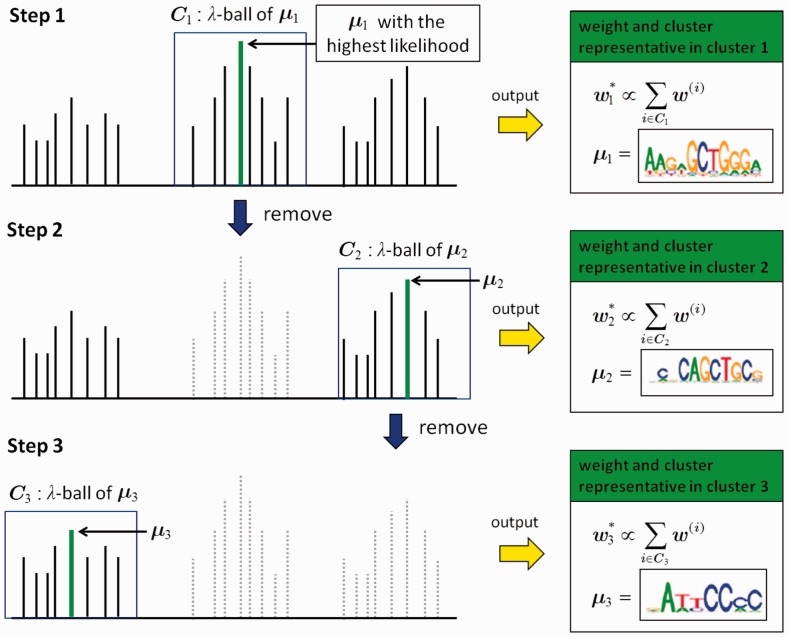
A schematic illustration of the post-processing process

RPMCMC produces many redundant outputs with slight variations. We reduce the redundancy by grouping the outputs into *g* clusters, $C_{1},\ldots ,C_{g}$, based on the dissimilarity of the sampled PPMs. The procedure is as follows (see Fig. 3 for a schematic illustration):

- Samples of size p=M×N are arranged as $\eta =\{x^{\left(1\right)},\ldots ,x^{\left(p\right)}\}$ by sorting realized values of the likelihood [Equation (1)] in decreasing order.
- Set λ>0, a threshold for the within-cluster variability.
- Set *k* = 1 and repeat (a)-(d) until no samples are left:
  - Initiate the *k*th cluster $C_{k}=\{x^{\left(1\right)}\}$ by a singleton of the sample that is ranked first in η. Let $\mu_{k}=x^{\left(1\right)}$ be the cluster representative.
  - Collect all samples satisfying the condition $D(\Theta^{\left(1\right)},\Theta^{\left(i\right)})\leq \lambda$ where $\Theta^{\left(i\right)}$ denotes the PPM of $x^{\left(i\right)}$. These samples are integrated into cluster *C_k_*; $C_{k}=\{x^{\left(i\right)}|D(\Theta^{\left(1\right)},\Theta^{\left(i\right)})\leq \lambda ,i=1,\ldots ,p\}$.
  - Discard the collected samples in *C_k_* from the ordered sequence; $\eta \leftarrow \eta \setminus C_{k}$. Let *p* be the length of η and rearrange η according to the likelihood values.
  - If η is empty, terminate the computation. Otherwise, let k←k+1 and go back to step (a).

The method operates with a single input parameter λ that controls the number *g* of clusters. Samples within D ≤ λ are assigned to the cluster representative μ*_k_*, which is the one to achieve the highest likelihood within the *k*th cluster members.

Denote the p=M×N samples and their importance weights by ${\left\{x^{\left(i\right)},w^{\left(i\right)}\right\}}_{i=1}^{p}$. With the *g* reduced samples $\left\{\mu_{1},\ldots ,\mu_{g}\right\}$, we define an approximated posterior distribution by

$$\hat{p}(x|S)\propto \sum_{k=1}^{g}I(x=\mu_{k})w_{k}^{*},w_{k}^{*}\propto \sum_{i\in C_{k}}w^{\left(i\right)}.$$

This is a mixture of the *g* probability mass functions $I(x=\mu_{k})$ at μ*_k_*. Mixing rate $w_{k}^{*}$ is the sum of the importance weights associated with the corresponding cluster *C_k_*. PPMs and the motif start sites in $\left\{\mu_{1},\ldots ,\mu_{g}\right\}$ are of primary interest for motif discovery. We generate a ranked list of the reduced discovered motifs, which are ordered according to the weights $w_{k}^{*}$.

### 2.5 Performance evaluation

We report the performance of several motif discovery algorithms on two types of data: (i) promoter sequences into which strings generated from PPMs in the JASPAR CORE database are planted and (ii) 228 TF ChIP-seq datasets of the ENCODE project. We evaluate the performance for each type of data as follows:

(i)  Given the nucleotide positions of known and predicted motifs, recall [sensitivity (SN)] and precision [positive predicted value (PPV)] are evaluated at a nucleotide level. These criteria have commonly been used, for instance in Tompa *et* *al*. (2005) (we use the abbreviations SN and PPV according to convention). For given *J* known motifs, we define slightly modified SN and PPV for the evaluation of multiple output motifs.
  Let *p_j_* be the output that achieves the most overlapping predicted sites with the *j*th known motif among the *g* outputs (if there are two or more outputs having the same number of overlapped nucleotides, the one with the higher rank given by a motif finder is chosen). Then, the recall and the precision are computed as

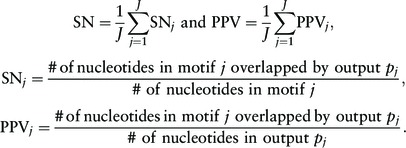

A low SN statistic indicates the lack of ability to discover planted multiple motifs and a low PPV statistic can be a signal for less identification accuracy, for instance the occurrence of over- or under-estimates of the planted motif regions.
(ii)  From contiguous segments around the transcription factor binding sites (TFBSs) of the primary TF in each dataset, we obtain a list of cofactor interacting motifs and their annotations that are implicated in the regulatory module of the primary TF. To identify the cooperative cofactors of the primary TF, each predicted motif (PPM) is matched to JASPAR CORE motifs by using the TOMTOM program (Gupta *et* *al*., 2007). For a given predicted PPM, TOMTOM outputs the matching scores to all annotated TFBSs (the name of TFs) in JASPAR with the statistical significances (*E*-values). For each algorithm, a diversity of the discovered motifs is evaluated with the number of known motifs in JASPAR CORE that are matched significantly to the produced PPMs with the acceptable level of significance at *E*-value less than 0.05.
  In addition, we use the log-likelihood ratio (LLR) to evaluate *K*-mer binding sites of a predicted motif:
  $$\text{LLR}(U,K)=\sum_{k=1}^{K}\sum_{\sigma \in \{a,c,g,t\}}n\prime f_{k,\sigma }\log\left(\frac{f_{k,\sigma }}{b_{\sigma }}\right),$$
  where $f_{\sigma ,k}$ (σ∈{a,c,g,t}, k∈{1,…,K}) is the relative frequency of nucleotides at each position in a predicted site, $b={\left(b_{a},b_{c},b_{g},b_{t}\right)}^{T}$ is the relative frequency of nucleotides of the background. The output consists of n′ motif subsequences. A higher LLR indicates a better likeliness of the *K*-mer instances to be a motif in terms of a combined characterization on the degree of overrepresentation relative to the background and the total information content.

## 3 Results and discussion

### 3.1 Synthetic promoter sequence

The performance of RPMCMC was tested on synthetic datasets against two ChIP-tailored algorithms, DREME and Hegma, and a classical algorithm, Weeder. The datasets were derived from non-redundant sets of randomly selected n∈{300,600,1200,2500,5000} promoter sequences obtained from UCSC.hg19 with two different kinds; one composed of fixed-length sequences of 1000 bp and the other of variable-length sequences varied between 200 and 2000 bp. Oligomers generated by randomly chosen 10 JASPAR CORE PPM collections were planted into randomly selected start sites, so that each sequence has eight motifs on average. For each data size *n*, we prepared 20 different sequence sets. With this ground truth, we measured the change in recall and precision. All parameters of RPMCMC and the specified Weeder options are listed in Table 1. For DREME and Hegma, we employed the default parameters. The parameters of RPMCMC were empirically chosen.

**Table 1.** Default parameters of RPMCMC and Weeder options that were used in all experiments

RPMCMC	
Parameter	Value
Prior on *z_i_*	γ=0.755
Max/min motif width	$K_{\min}=8,K_{\max}=15$
Dirichlet priors	$\alpha_{\sigma }=1,\beta_{k,\sigma }=1$
No. of replicas	*M* = 50
No. of MCMC iterations	*N* = 520
Burn-in period (fixed)	N≤20
Repulsive force severity	$\beta =10\times \sum_{i}z_{i}$
Motif clustering	λ=0.3
Gap penalty	*c* = 0.3

Weeder	
Option	Value

Species code	HS
Analysis type	Medium

Hegma and DREME were executed using the default settings.

**Fig. 4.**
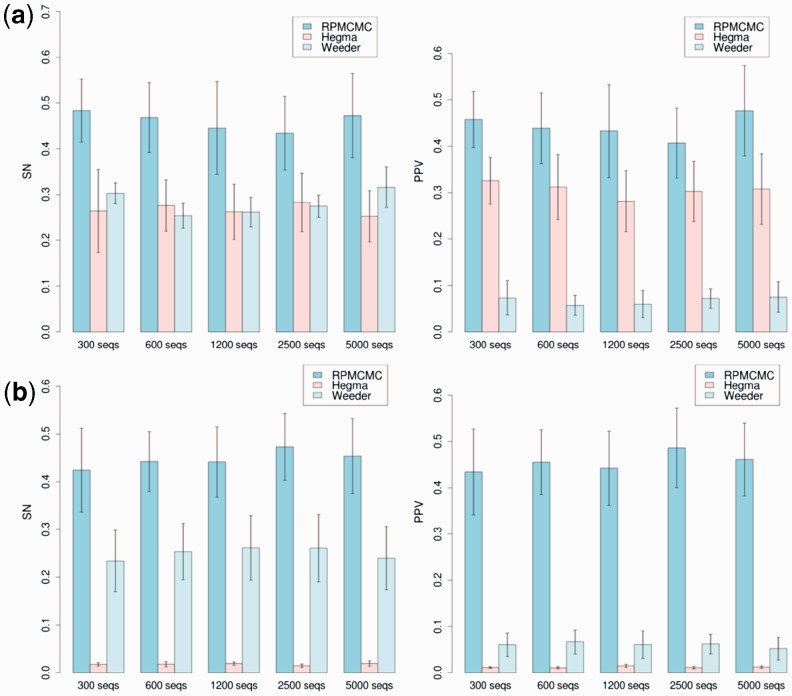
Performance comparison among RPMCMC, Hegma and Weeder on synthetic datasets: (**a**) fixed-length sequence sets and (**b**) variable-length sequence sets. Motifs were generated according to the JASPAR CORE PPM collection and were inserted randomly into a set of promoter sequences. SN (left) and PPV (right) values of each method are plotted against the varying sequence sizes, n∈{300,600,1200,2500,5000}

Figure 4(a) summarizes the SN and PPV values as a function of *n* for RPMCMC, Hegma and Weeder. DREME was removed from this figure because there was no way of calculating SN and PPV due to the lack of outputs on motif sites in the distributed program. The numbers of outputs from RPMCMC, Hegma and Weeder were 85.7, 214.76 and 13.3 on average, respectively. It can be seen that RPMCMC outperformed the other methods. For the fixed-length datasets, RPMCMC delivered SN values around 1.7 times higher than those of the other two methods. The PPVs of RPMCMC were around 1.5 times higher than those of Hegma. As shown in Figure 4(b), the results on the variable-length datasets were similar to those on the fixed-length datasets except that the performance of Hegma was significantly degraded.

We analyzed the cause of the observed low SN and PPV statistics for Hegma and Weeder, as illustrated with the results on the fixed-length datasets. It was found that Hegma has a strong tendency to divide planted regions of a motif into a few different predicted motifs. Such incorrectly fragmented outputs acted to increase PPV slightly but resulted in the observed low SN. A distinctive characteristic of Weeder is the fairly low PPV, whereas several comparative studies reported Weeder to be one of the best performing algorithms among early motif finders (Tompa *et* *al*., 2005). A region predicted by Weeder tends to include not only a planted motif region but also many background regions. RPMCMC could achieve much higher SN and PPV than the others could.

**Fig. 5.**
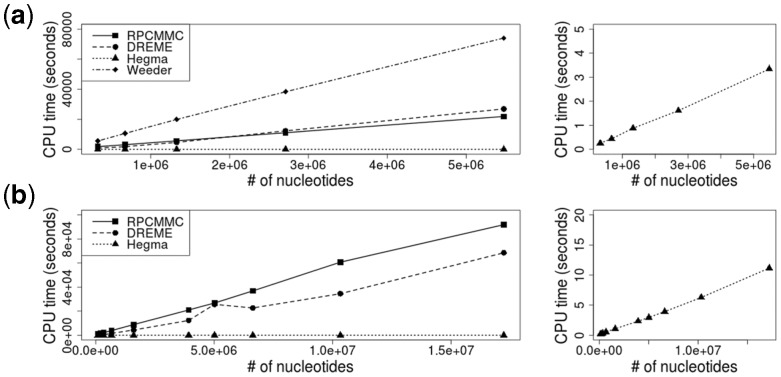
Computational efficiency of RPMCMC, Hegma, DREME and Weeder. (**a**) The synthetic promoter sequence and (**b**) the ChIP-seq datasets, shown as a function of the number of nucleotides. The vertical axis indicates CPU times. The right figure is an enlarged display of the left figure to make clear the computation time of Hegma

Figure 5(a) gives the computation time for each method. RPMCMC was implemented in C++. We used the C programs for DREME, Hegma and Weeder, which are available on the authors’ websites. All the tests were conducted on Intel^®^ Xeon Phi^™^coprocessors with 61-core CPUs and 48 GB of main memory. In terms of computation efficiency, Hegma outperformed the others and RPMCMC was comparable to DREME. In particular, the computation times of RPMCMC and DREME were about a 10000th those of Hegma. RPMCMC would sustain an acceptable level of computation time, and furthermore, it might be possible to render the algorithm more efficient. The bottleneck in RPMCMC is in the process of calculating the posterior probabilities of the motif start sites *u_i_* (see details in Supplementary Method S1): with a given PPM, $K\times \sum_{i}2(L_{i}-K+1)$ times calculations were necessary to perform in every iteration over all possible *K*-mer consecutive subsequences in *S*. This process can fully be parallelized into independent processing elements. Alternatively, we could use a branch-and-bound technique as in STEME that effectively prunes subsequences with negligibly low probabilities.

**Fig. 6.**
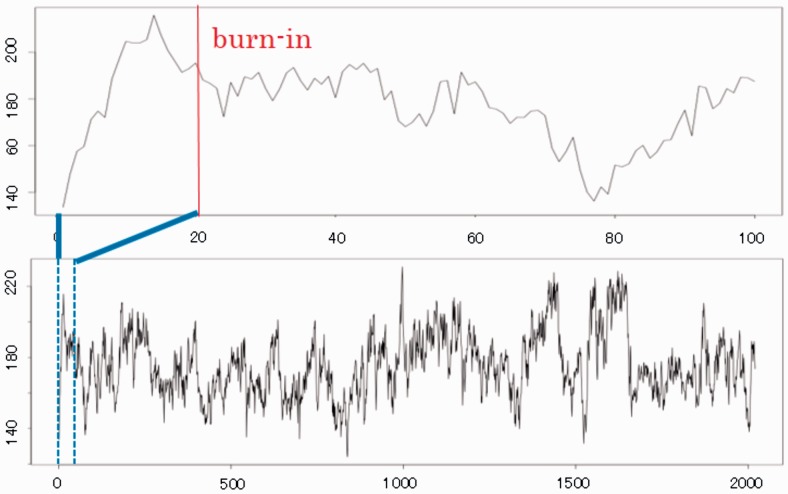
Series of the likelihood values in RPMCMC for a synthetic dataset with 300 sequences. Default burn-in is set at 20 steps (vertical line in upper plot)

We remark on the difficulty in detecting the burn-in time for RPMCMC. An initial portion of the Markov chain samples should be discarded because the chain approaches its stationary distribution (Cowles and Carlin, 1996) following a sufficient burn-in period. Figure 6 shows the process of evolving the likelihood during a RPMCMC run. The series of the likelihood values remained instable, which indicates a fairly slow mixing of the Markov chain because the target distribution was inherently multimodal and the parallel interacting chains switched their target local modes successively. In general, it is difficult to deal with a diagnostic of burn-in periods that looks for multimodality of the posterior distribution. At the current moment, we do not have a specific idea other than an obvious approach of giving as long as possible for a trial move.

### 3.2 ENCODE ChIP-seq datasets

Using RPMCMC with the default parameters given in Table 1, we predicted the cofactor motifs of the primary TF for each of the 228 datasets of ChIP-seq experiments in the ENCODE project (The ENCODE Project Consortium, 2012). FASTA files were produced by clipping the sequences of UCSC.hg19 at the locations recorded in SYDH TFBS narrowPeak files (available from NCBI’s Gene Expression Omnibus using the accession number GSE31477). We removed datasets that had only a few sequences after removing fragments with lengths less than 200 or more than 500 from the obtained FASTA files. Also, we removed datasets which have more than one percent of sequences including blacklist regions reported on https://sites.google.com/site/anshulkundaje/projects/blacklists. In this way, we obtained the 228 datasets from the total of 359 datasets. The numbers of the input sequences ranged from 205 to 49 211. RPMCMC produced 51–149 output motifs for each dataset. A discovered motif, for instance $\left\{U_{k},\Theta_{k}\right\}$ in μ*_k_*, was regarded as being significantly enriched if it appeared in 5% or more of the input sequences, i.e. $\sum_{i=1}^{n}z_{i}/n\geq 0.05$. At the acceptable level of significance on the TOMTOM’s *E*-values ≤0.05, approximately 15 significantly enriched outputs on average could have correspondence to one of the experimentally validated TFBSs in JASPAR CORE. In Supplementary Data S1, we provide the lists of *de novo* cofactor motifs for all TF-ChIP datasets with the results of JASPAR annotations.

In the experiments, Hegma produced a far greater number of outputs (1081 outputs on average over all datasets) than RPMCMC (110 outputs) and DREME (49 outputs). The outputs of Hegma possibly included many redundant motifs. Removing motifs with $\sum_{i=1}^{n}z_{i}/n<0.05$ from the total outputs, the average numbers of outputs of Hegma, RPMCMC and DREME dropped to 24, 110 and 33, respectively.

The computation times of each algorithm for 10 selected datasets including the smallest and the largest dataset are shown in Figure 5(b). Compared with the experiment with the synthetic datasets, the computation times of RPMCMC were a little inferior to those of DREME for the ChIP-seq datasets. RPMCMC would still sustain an acceptable level of computation time. As discussed in the previous subsection, the current implementation of the RPMCMC algorithm is yet to be optimized for speed.

**Fig. 7.**
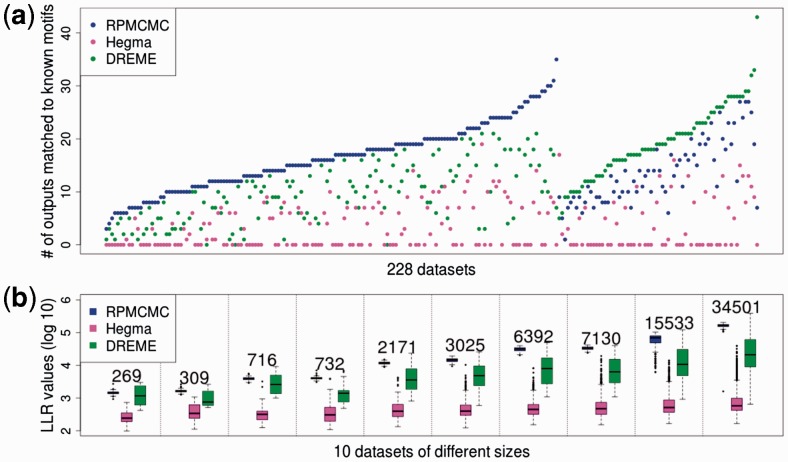
Comparison of RPMCMC with Hegma and DREME on the 228 ENCODE datasets. (**a**) The number of motifs in JASPAR CORE that were matched to outputs of each algorithm for each of the 228 datasets (blue: RPMCMC; magenta: Hegma; green: DREME). The datasets are arranged by gathering together the subsets with which each method achieved the most matching to JASPAR. (**b**) The LLR values of the predicted sites are shown across arbitrary-chosen 10 datasets with different sizes ($\log_{10}$). Each number on the box indicates the number of sequences in each dataset

As shown in Figure 7(a), the numbers of known motifs significantly matched to the outputs of RPMCMC (*E*-values ≤0.05) were larger than those of Hegma and DREME for 74% of the 228 datasets. Although RPMCMC produced the largest numbers of outputs among the three methods, the LLR values of the discovered motifs of RPMCMC were much higher than those of the others as in Figure 7(b). This indicates that RPMCMC has a great potential to mine many reliable diverse motifs that are undetected by the existing methods.

Table 2 shows 15 cofactors that were predicted by RPMCMC on a ChIP dataset (wgEncodeSydhTfbsHepg2Nrf1IggrabPk) in which the binding sites of NRF1 were studied in HepG2. The binding sites of RPN4 and USF1 were detected only by RPMCMC. It was reported that both RPN4 and NRF1 are involved in the same proteasome activity (Radhakrishnan *et* *al*., 2010; Xu *et* *al*., 2011), and the interaction of USF1 and Nrf1 is involved in the transcriptional regulation of FMP1 gene (Prasad and Singh, 2008).

**Table 2:** A list of 16 predicted motifs obtained by RPMCMC that are implicated in the transcriptional module of NRF1 in HepG2

Predicted motif	E-value	Ranking	P/A		
			Hegma	DREME
SP1	$2.71\times 10^{-4}$	1	P	P
EGR1	$1.09\times 10^{-3}$	1	P	P
SP2	$1.10\times 10^{-3}$	1	P	P
KLF5	$3.75\times 10^{-3}$	1	P	P
NRF1	$3.80\times 10^{-9}$	2	P	P
FUS3	$5.07\times 10^{-3}$	2	P	P
E2F4	$4.52\times 10^{-2}$	45	A	P
REST	$3.92\times 10^{-3}$	47	A	A
GABPA	$1.57\times 10^{-2}$	51	A	A
DAF-12	$1.41\times 10^{-2}$	56	A	A
MET31	$1.56\times 10^{-2}$	62	A	A
RPN4	$3.49\times 10^{-2}$	62	A	A
TYE7	$1.43\times 10^{-3}$	70	A	A
PIF5	$4.35\times 10^{-2}$	70	A	A
USF1	$4.56\times 10^{-2}$	70	A	A
RDS1	$3.37\times 10^{-2}$	71	A	A

NRF1 is the ChIPed TF and the rest are the predicted cofactors. All motifs, which could be annotated at *E*-value ≤ 0.05 according to JASPAR, are shown with the *E*-values of TOMTOM (second column) and the ranking by RPMCMC (third column). The last two columns indicate the presence (P) or absence (A) of the motif in the outputs of Hegma and DREME, respectively.

**Fig. 8.**
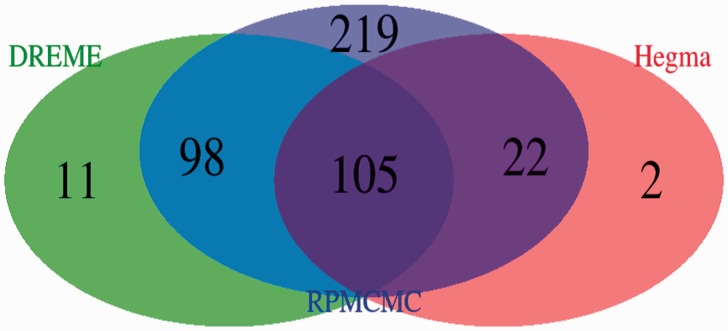
Venn diagram for total numbers of significantly annotated motifs over all the 228 datasets, reported by RPMCMC, Hegma and DREME

Figure 8 summarizes the detection ability to discover diverse motifs based on a Venn diagram of all matching motifs produced from the analyses of the 228 datasets. The outputs of RPMCMC contained almost all of the outputs of DREME and Hegma, and, notably, 219 annotated cofactors were uniquely discovered by RPMCMC.

## 4 Concluding remarks

In the motif discovery problem, the direct use of a Gibbs sampling method revealed an inability to find latent diverse motifs even in a fairly small number of input sequences. In the application for only 300 input sequences, all simulations with different initializations became trapped in the AT-rich motifs, which are of little significance in practice. This highlighted a critical drawback of the Gibbs sampling methods. The same is true for the EM algorithm. Because biological sequences generally contain rather diverse conserved patterns, which are sometimes biologically meaningless, the posterior distribution exhibits a very complex landscape as it includes many locally high probability regions. Our view is that solving this problem is the essence of improving the accuracy of motif discovery. Motivated by this, we presented a new motif discovery method called RPMCMC, which is a parallel variant of the widely used Gibbs motif samplers. The rather simple idea is to run the Gibbs motif samplers in parallel by making use of the repulsive force on different samplers. With all-at-once sampling, we could discover diverse motifs by which the parallel samplers divide their responsibility in the overall search region.

As another contribution, we provided a list of predicted cofactor motifs that were overrepresented in the 228 ENCODE ChIP-seq datasets. RPMCMC can potentially mine promising annotated motifs which other word-count methods fail to find. To narrow down things to truly functional cofactor sets, it is necessary to conduct further validation experiments.

## Supplementary Material

Supplementary Data

## References

[btv017-B1] Bailey T. (2011) DREME: motif discovery in transcription factor ChIP-seq data. Bioinformatics, 27, 1653–1659. 21543442 10.1093/bioinformatics/btr261 PMC3106199

[btv017-B2] Bailey T. Elkan C. (1994) Fitting a mixture model by expectation maximization to discover motifs in biopolymers. In Proceedings of the Second International Conference on Intelligent Systems for Molecular Biology, pp. 28–36, AAAI Press, Menlo Park, California. 7584402

[btv017-B3] Bailey T. (2010) The value of position-specific priors in motif discovery using MEME. BMC Bioinformatics, 11, 179. 20380693 10.1186/1471-2105-11-179 PMC2868008

[btv017-B4] Cowles M. Carlin P. (1996) Markov chain Monte Carlo convergence diagnostics: a comparative review. J. Am. Stat. Assoc., 91, 883–904.

[btv017-B5] da Fonseca P. (2008) Efficient representation and P-value computation for high-order Markov motifs. Bioinformatics, 24, i160–i166. 18689819 10.1093/bioinformatics/btn282

[btv017-B7] Goi C. (2013) Cell-type and transcription factor specific enrichment of transcriptional cofactor motifs in ENCODE ChIP-seq data. BMC Genomics, 14(Suppl. 5), S2. 24564528 10.1186/1471-2164-14-S5-S2 PMC3852067

[btv017-B8] Green P. (1995) Reversible jump Markov chain Monte Carlo computation and Bayesian model determination. Biometrika, 82, 711–732.

[btv017-B9] Gray F. (1947) Pulse code communication. U.S. Patent 2632058.

[btv017-B10] Gupta S. (2007) Quantifying similarity between motifs. Genome Biol., 8, R24. 17324271 10.1186/gb-2007-8-2-r24 PMC1852410

[btv017-B11] Hughes J. (2000) Computational identification of cis-regulatory elements associated with groups of functionally related genes in *Saccharomyces cerevisiae*. J. Mol. Biol., 296, 1205–1214. 10698627 10.1006/jmbi.2000.3519

[btv017-B12] Ichonose N. (2012) Large-scale motif discovery using DNA Gray code and equiprobable oligomers. Bioinformatics, 28, 25–31. 22057160 10.1093/bioinformatics/btr606 PMC3244767

[btv017-B13] Lawrence C. (1993) Detecting subtle sequence signals: a Gibbs sampling strategy for multiple alignment. Science, 262, 208–214. 8211139 10.1126/science.8211139

[btv017-B14] Neal R. (2003) Slice sampling. Ann. Stat., 31, 705–767.

[btv017-B15] Pavesi G. (2001) An algorithm for finding signals of unknown length in DNA sequences. Bioinformatics, 17, S208–S214. 10.1093/bioinformatics/17.suppl_1.s207 11473011

[btv017-B16] Prasad S. Singh K. (2008) Interaction of USF1/USF2 and alpha-Pal/Nrf1 to Fmr-1 promoter increases in mouse brain during aging. Biochem. Biophys. Res. Commun., 376, 347–351. 18782566 10.1016/j.bbrc.2008.08.155

[btv017-B17] Radhakrishnan S. (2010) Transcription factor Nrf1 mediates the proteasome recovery pathway after proteasome inhibition in mammalian cells. Mol. Cell., 38, 17–28. 20385086 10.1016/j.molcel.2010.02.029 PMC2874685

[btv017-B18] Reid J. Wernisch L. (2011) STEME: efficient EM to find motifs in large data sets. Nucleic Acids Res., 39, e126. 21785132 10.1093/nar/gkr574 PMC3185442

[btv017-B19] Sandelin A. (2004) JASPAR: an open-access database for eukaryotic transcription factor binding profiles. Nucleic Acids Res., 32(Database issue), D91–D94. 14681366 10.1093/nar/gkh012 PMC308747

[btv017-B20] Sharov A. Ko M. (2009) Exhaustive search for over-represented DNA sequence motifs with CisFinder. DNA Res., 16, 261–273. 19740934 10.1093/dnares/dsp014 PMC2762409

[btv017-B22] Smith A. (2005) Mining ChIP-chip data for transcription factor and cofactor binding sites. Bioinformatics, 21, 403–412. 10.1093/bioinformatics/bti1043 15961485

[btv017-B23] The ENCODE Project Consortium (2012) An integrated encyclopedia of DNA elements in the human genome, Nature, 489**,** 57–74. 22955616 10.1038/nature11247 PMC3439153

[btv017-B24] Tompa M. (2005) Assessing computational tools for the discovery of transcription factor binding sites. Nat. Biotechnol.*,* 23**,** 137–144. 15637633 10.1038/nbt1053

[btv017-B25] Xu H. (2011) The CCAAT box-binding transcription factor NF-Y regulates basal expression of human proteasome genes. Biochim. Biophys. Acta., 1823**,** 818–825. 22285817 10.1016/j.bbamcr.2012.01.002

[btv017-B26] Wingender E. (1995) TRANSFAC: a database on transcription factors and their DNA binding sites. Nucleic Acids Res., 24, 238–241. 8594589 10.1093/nar/24.1.238 PMC145586

[btv017-B27] Workman C. Stormo G. (2000) ANN-Spec: a method for discovering transcription factor binding sites with improved specificity. Pac. Symp. Biocomput., 5, 467–478. 10902194 10.1142/9789814447331_0044

