# Supplementary material for: Repulsive parallel MCMC algorithm for discovering diverse motifs from large sequence sets
Source: Bioinformatics. 2015 Jan 11;31(10):1561–8. doi: 10.1093/bioinformatics/btv017 (PMC4426842; doi:10.1093/bioinformatics/btv017)
Supplement: Supplementary Data [file supp_31_10_1561__index.html]

Repulsive parallel MCMC algorithm for discovering diverse motifs from large sequence sets — Repulsive parallel MCMC algorithm for discovering diverse motifs from large sequence sets — Repulsive parallel MCMC algorithm for discovering diverse motifs from large sequence sets — Supplementary Data 

# Repulsive parallel MCMC algorithm for discovering diverse motifs from large sequence sets

## Supplementary Data

files

**Files in this Data Supplement:**

- Supplementary Data - pdf file
