# Supplementary material for: Repulsive parallel MCMC algorithm for discovering diverse motifs from large sequence sets
Source: Bioinformatics. 2015 Jan 11;31(10):1561–8. doi: 10.1093/bioinformatics/btv017 (PMC4426842; doi:10.1093/bioinformatics/btv017)
Supplement: Supplementary Data [file supp_btv017_revised-supplementary.pdf]

Supplementary Data S1: List of predicted cofactor motifs for 228 ENCODE ChIP-seq datasets

The data are available at http://daweb.ism.ac.jp/yoshidalab/motif.

Supplementary Method S1: RPMCMC Procedure

The RPMCMC algorithm renews each of the M replicas, $x_m = \{U^m, Z^m, K^m, \Theta^m, \theta_0^m\}$ $(m = 1, \ldots, M)$ by alternatively drawing a sample from the full conditional distribution of the extended target (Eq. 2) while fixing all others at the current values. The procedure for sampling U^m, Z^m, and θ_0^m is the same as that for the conventional Gibbs motif sampler because these components are independent of the repulsive force function. The procedure for updating Θ and K involves a reversible-jump MCMC algorithm (Green, 1995) and a slice sampler (Neal, 2003), which are used to treat the varying-width PPM and the repulsive force acting upon the PPMs. Each sampling procedure is detailed while omitting the replica index $(m = 1, \ldots, M)$ except for the description of Θ and K:

Motif start site: The conditional posterior probability of u_i $(i = 1, \ldots, n)$ given the others is

$$p(u_i|x \setminus \{u_i\}, S) \propto \prod_{k=1}^{K} \prod_{\sigma\in(a,c,g,t)} \left(\frac{\theta_{k,\sigma}}{\theta_{0,\sigma}}\right)^{I(\sigma=s_{i,u_i+k-1})}, \quad u_i \in \{1, \ldots, L_i - K + 1, L_i + 1, \ldots, 2L_i - K + 1\}.$$

We draw a value of u_i after normalizing the above so as to sum up to one over $u_i \in \{1, \ldots, L_i - K + 1, L_i + 1, \ldots, 2L_i - K + 1\}$. The process of sampling u_i $(i = 1, \ldots, n)$ can be fully-parallelized into n independent calculations. We run this with an OpenMP implementation of multi-threading.

Motif presence indicator: The conditional posterior of each z_i $(i = 1, \ldots, n)$ is

$$p(z_i|x \setminus \{z_i\}, S) \propto \begin{cases} \gamma^K \prod_{k=1}^{K} \prod_{\sigma} (\frac{\theta_{k,\sigma}}{\theta_{0,\sigma}})^{I(s_{i,u_i+k-1}=\sigma)} & (z_i = 1) \\ 1 - \gamma^K & (z_i = 0) \end{cases}$$

This process was also parallelized into n independent threads in the OpenMP implementation.

PPM: For the mth replica, the kth column θ_k^m of PPM is updated by drawing a sample from the following full conditional distribution:

$$\theta_k^m \sim p(\theta_k^m|x_m \setminus \{\theta_k^m\}, S) \propto \prod_{\sigma\in\{a,c,g,t\}} \theta_{k,\sigma}^{m\ \sum_{i=1}^{n} I(s_{i,u_i^m+k-1}=\sigma)+\beta_{k,\sigma}-1} \exp(\min_{j:j\neq m} D(\Theta^m, \Theta^j)),$$

for $k = 1, \ldots, K$.

Here, it is impossible to obtain samples directly from this analytically intractable distribution which is the product of a Dirichlet density function and the repulsive function. The current version of RPMCMC uses a slice sampler.

Without loss of generality, we let $\theta_{k,t}^m = 1 - \theta_{k,a}^m - \theta_{k,c}^m - \theta_{k,g}^m$ be fixed. In the slice sampler, for each $\sigma \in a, g, c$, a new $\theta_{k,\sigma}^m$ is sampled from the uniform distribution in the range between $\theta_{\min}$ and $\theta_{\max}$ as follows:

(i) Generate ϵ from the uniform distribution in the range $[0, 1]$.

(ii) Find $\theta_{\max}$ such that $p(\theta_{\max}|x \setminus \theta_{k,\sigma}^m, S) = \epsilon \times p(\theta_{k,\sigma}^m|x \setminus \theta_{k,\sigma}^m, S)$ by incrementing $\theta_{\max}$ from the current $\theta_{k,\sigma}^m$ by a small value δ.

(iii) Find $\theta_{\min}$ such that $p(\theta_{\min}|x \setminus \theta_{k,\sigma}^m, S) = \epsilon \times p(\theta_{k,\sigma}^m|x \setminus \theta_{k,\sigma}^m, S)$ by decrementing $\theta_{\min}$ from $\theta_{k,\sigma}^m$ by δ.

(iv) Generate the new $\theta_{k,\sigma}^m$ from the uniform distribution in the range $[\theta_{\min}, \theta_{\max}]$.

Background probability: θ_0 is updated by drawing a Dirichlet random variable from the full conditional distribution

$$p(\theta_0|x \setminus \{\theta_0\}, S) \propto \prod_{\sigma \in \{a,c,g,t\}} \theta_{0,\sigma}^{\sum_{i=1}^{n}(\sum_{j=1}^{2L_i} I(s_{i,j}=\sigma) - \sum_{k=1}^{K} I(s_{i,u_i+k-1}=\sigma)) + \beta_{0,\sigma} - 1}.$$

Motif length: Θ and K are updated by the reversible jump MCMC algorithm (Green, 1995), as shown in the following procedure:

Suppose that the currently obtained Θ has the width $K = k$. To renew $\{\Theta, k\}$ to $\{\Theta^*, k^*\}$ at a step, we first generate a candidate according to the proposal distribution:

$$q(\Theta^*, K^* = k^* \mid \Theta, K = k) = \begin{cases} q_{lc} & k^* = k - 1 : \Theta^* = \Theta_{2:k} \\ q_{rc} & k^* = k - 1 : \Theta^* = \Theta_{1:k-1} \\ q_0 & k^* = k : \Theta^* = \Theta \\ q_{le} & k^* = k + 1 : \Theta^* = (\theta^*, \Theta) \\ q_{re} & k^* = k + 1 : \Theta^* = (\Theta, \theta^{**}) \end{cases}$$

The first two transitions indicate the contraction proposals that drop the first and last columns of $\Theta = (\theta_1, \Theta_{2:k}) = (\Theta_{1:k-1}, \theta_k)$, respectively, from the current Θ. The third transition is to retain the current state. The last two transitions expand the size of the current PPM to $K^* = k + 1$ by adding the new components, θ^* and θ^{**}, to the leftmost and rightmost columns of Θ. Conditioned by Z, U, and $K = k$, these additional components are given by the frequencies of each nucleotide at the first and last elements of the currently occupied motif region:

$$\theta_\sigma^* = \frac{\sum_{i=1}^n I(s_{i,u_i-1} = \sigma)}{\sum_{\sigma'} \sum_{i=1}^n I(s_{i,u_i-1} = \sigma')} \quad \text{and} \quad \theta_\sigma^{**} = \frac{\sum_{i=1}^n I(s_{i,u_i+k} = \sigma)}{\sum_{\sigma'} \sum_{i=1}^n I(s_{i,u_i+k} = \sigma')}.$$

After one of the move types is chosen according to the probabilities $q_{lc}, q_{rc}, q_0, q_{le}, q_{re}$, we determine the acceptance or rejection according to the probability,

$$\alpha(\Theta^*) = \min\left(1, \frac{p(U, Z, K = k^*, \Theta^*, \theta_0|S) q(\Theta, K = k|\Theta, K = k)}{p(U, Z, K = k, \Theta, \theta_0|S) q(\Theta^*, K = k^*|\Theta^*, K = k^*)}\right).$$

If accepted, $\Theta^* \to \Theta$, and otherwise $\Theta \to \Theta$.

References

1. Green,P. (1995) Reversible jump Markov chain Monte Carlo computation and Bayesian model determination. *Biometrika*, 82, 711-732.

2. Neal,R. (2003) Slice sampling. *Ann. Stat.*, 31, 705-767.
